# NaCl Induces Flavonoid Biosynthesis through a Putative Novel Pathway in Post-harvest Ginkgo Leaves

**DOI:** 10.3389/fpls.2017.00920

**Published:** 2017-06-12

**Authors:** Jun Ni, Juan Hao, Zhifang Jiang, Xiaori Zhan, Lixiang Dong, Xiuli Yang, Zhehang Sun, Wenya Xu, Zhikun Wang, Maojun Xu

**Affiliations:** ^1^Key Laboratory of Hangzhou City for Quality and Safety of Agricultural Products, College of Life and Environmental Sciences, Hangzhou Normal UniversityHangzhou, China; ^2^Zhejiang Provincial Key Laboratory for Genetic Improvement and Quality Control of Medicinal Plants, Hangzhou Normal UniversityHangzhou, China; ^3^Department of Molecular Cellular and Developmental Biology, College of Letters and Science, University of California, Santa BarbaraSanta Barbara, CA, United States

**Keywords:** flavonoid biosynthesis, gene expression, Ginkgo leaves, NaCl, post-harvest, ultraviolet-B

## Abstract

The flavonoids in the extracts of Ginkgo leaves have been shown to have great medical value: thus, a method to increase the flavonoid contents in these extracts is of significant importance for human health. In the present study, we investigated the changes in flavonoid contents and the corresponding gene expression levels in post-harvest Ginkgo leaves after various treatments. We found that both ultraviolet-B and NaCl treatment induced flavonoid accumulation. However, gene expression analysis showed that the increases in flavonoid contents were achieved by different pathways. Furthermore, post-harvest Ginkgo leaves responded differently to NaCl treatment compared with naturally grown leaves in both flavonoid contents and corresponding gene expression. In addition, combined treatment with ultraviolet-B and NaCl did not further increase the flavonoid contents compared with ultraviolet-B or NaCl treatment alone. Our results indicate the existence of a novel mechanism in response to NaCl treatment in post-harvest Ginkgo leaves, and provide a technique to increase flavonoid content in the pharmaceutical industry.

## Introduction

Flavonoids bbb are ccc a major class of plant secondary metabolic products and are widely distributed in the plant kingdom (Winkel-Shirley, 2001a,b). Flavonoids have a multitude of biological functions, including defense against phytopathogens and herbivores, they act as ultraviolet (UV) filters for tissue protection, attract insect pollinators by producing colorful anthocyanins, are involved in pollen germination and biological communication in the rhizosphere, regulate auxin transport and catabolism, and most importantly, act as antioxidants, inhibiting the generation of reactive oxygen species (ROS), which occurs in stressed plants (Mouradov and Spangenberg, 2014). Interestingly, the capacity of flavonoids to act as antioxidants to protect the body against ROS has also been proven to have benefits in human health such as cancers, cardiovascular, and metabolic diseases (Lu et al., 2013). Due to the commercial significance and pharmacological activities of flavonoids, they are referred as “nutraceuticals” (Tapas et al., 2008; Santhosh and Suriyanarayanan, 2014).

The flavonoid biosynthesis pathway in the model plant *Arabidopsis thaliana* has been extensively characterized. Genes responsible for the formation of flavonoid structures and subsequent modifications have been characterized using functional genomic approaches (Shirley et al., 1995; Saito et al., 2013). Moreover, the biosynthesis of flavonoids is regulated by a variety of different developmental and environmental cues (Winkel-Shirley, 2002). Many external factors, such as light, temperature, wounding, nutrient status, drought stress, and pathogen infection, have been reported to affect flavonoid biosynthesis (Christie et al., 1994; Dixon and Paiva, 1995; Chalker-Scott, 1999; Carbone et al., 2009).

Ginkgo (*Ginkgo biloba* L.) has been cultivated for its medical properties for a long time, and extracts of Ginkgo leaves (EGB) are commonly used due to their broad range of pharmacological activities (Ward et al., 2002). Further research has revealed that the main bioactive constituents responsible for these pharmacological activities are flavonoids (Singh et al., 2008). Thus, it is of both biological and commercial importance to investigate the flavonoid biosynthesis pathway in Ginkgo leaves.

Since the cloning of chalcone synthase (*GbCHS*) gene (Pang et al., 2005), many genes related to flavonoid biosynthesis have been cloned and reported in Ginkgo, including *GbPAL* (Xu et al., 2008a), *GbCHI* (Cheng et al., 2010), *GbF3H* (Xu Y. et al., 2014), *GbFLS* (Xu et al., 2011), and *GbANS* (Xu et al., 2008b). Most of these genes are expressed throughout the plant, and regulated by different external and internal factors. In addition, a negative regulator of flavonoid synthesis, gene GbMYBF2, was also reported (Xu F. et al., 2014). These genes constitute a framework of flavonoid synthesis, and their expression levels can account for the changes in flavonoid contents in response to environmental stimuli in Ginkgo (Xu Y. et al., 2014).

The first step of EGB production in the pharmaceutical industry is the collection and storage of fresh Ginkgo leaves. Following separation of leaves from the Ginkgo trees, cells in the leaves are still alive and involved in the metabolism of flavonoids. Many attempts have been made to investigate flavonoid biosynthesis in Ginkgo cell lines (Xu et al., 2009, 2012). Previous research revealed that UV-B radiation is an effective inducer of flavonoid biosynthesis in post-harvest Ginkgo leaves (Sun et al., 2010). Are there any other factors which also affect flavonoid biosynthesis in post-harvest Ginkgo leaves? Does the combination of multiple factors further increase the concentration of flavonoids? These questions are both of scientific and commercial importance.

In this research, we examined the changes in flavonoid contents and the expression of corresponding genes in post-harvest Ginkgo leaves after various treatments. Our research indicated that UV-B and NaCl treatment induced the accumulation of flavonoids through different pathways. In addition, NaCl treatment triggered a novel method of increasing flavonoids in post-harvest Ginkgo leaves.

## Materials and Methods

### Plant Materials and Experimental Procedures

For the culture of post-harvest Ginkgo leaves, short shoots were harvested from Ginkgo trees aged approximately 15 years in Hangzhou Normal University, China (April and May, 2016). The samples were collected at a height of 3–4 m above the ground using a long reach chain saw in June 2016, and similar short shoots were chosen for further analysis. These short shoots were immediately transferred to Murashige and Skoog (MS, Gibco) solution (without sucrose) for culture in a growth room with continuous light (50 μmol∙m^-2^∙s^-1^) and constant temperature (25°C). Under this condition, the contamination was not observed for at least 24 h. The values of *F*v/*F*m and *F*n were measured with a fluorescence spectrophotometer (PAM-2100) and a portable photosynthesis system (LI-6400XT), respectively.

For the different treatments, cultured post-harvest Ginkgo leaves were recovered in MS solution for 12 h, then treated with UV-B (10 μmol∙m^-2^∙s^-1^), NaCl (200 mM), PEG (20%), CdCl_2_ (0.5 mM), and CuCl_2_ (0.5 mM) for 3 h. We used a UVB-313 lamp (Q-Lab) for UV-B treatment. The wavelength of this lamp ranged from 280 to 315 nm, with the strongest energy at 313 nm. For the NaCl treatment of Ginkgo trees, 2-year-old seedlings of uniform size were selected and planted in pots (34 cm^∗^23 cm, diameter^∗^height), containing medium composed of peat, yellow sand, and loam (1:1:2). These pots were placed outdoors and the trees were grown during 2016 growing season in Hangzhou. The experiment was carried out in the morning (June, 2016). For the NaCl treatment, we poured 3 L of 200 mM NaCl solution to the pots and waited for 3 h before harvest. The same volume of water was used as control treatment.

### Analysis of Flavonoids by High Performance Liquid Chromatography (HPLC) and Mass Spectra Analysis

The extraction and high performance liquid chromatography (HPLC) analysis of flavonoids of Ginkgo leaves were carried out as described before with mild modification (Tohge et al., 2005). Briefly, frozen leaves were extracted in 30 μl extraction solvent (methanol: acetate: H_2_O = 9:1:10) per 1 mg dry weight of tissues at 37°C 30 min. After centrifugation at 14000 × *g*, the supernatant was filtered through a 0.25 μm filter membrane. Ten microliters of supernatant was applied to waters HPLC e2695 series. HPLC was carried out on a XBridge C18 (Φ4.6 mm × 250 mm) at flow rate of 0.5 ml/min. Elution gradient with solvent A [CH_3_CN-H_2_O-TFA (10:90:0.1)] and solvent B [CH_3_CN-H_2_O-TFA (90:10:0.1)] and the following elution profile (0 min 100% A, 30 min 70% A, 32 min 0% A, 33 min 0% A, 35 min 100% A) using linear gradients in between the time points. We used rutin (R106912, Aladdin) and narcissoside (SMB00581, Sigma) standards for confirmation of flavonoids. The concentration of flavonoid standard solution was 1 mM. For the addition of standards, 20 μl of standard solution was added to 1 ml of EGB for HPLC analysis. PDA was used for detection of UV-visible absorption in the range of 190–510 nm. Flavonoids were detected at 360 nm.

The mass spectra analysis of flavonoids were carried out using a LCQ ion trap mass spectrometer (Finnigan MAT, San Jose, CA, United States) equipped with an ESI as described before (Dong et al., 2007). The mass spectrometer was connected to the HPLC system via an UV cell outlet. Helium was used as the buffer gas and nitrogen was used as the sheath gas. The arbitrary settings for collision energy in the ion trap and in-source collisional activation ranged from 20 to 30% and 10 to 20%, respectively. The heated metal capillary temperature was at 220°C. The electrospray voltage was at 5.0 kV. High-resolution mass spectrometry was performed using an Ultima 7.0 FTICR mass spectrometer (IonSpec, United States) with an ESI source both in the positive and negative ion mode. Probe heater temperature was at 100°C. Source heater temperature was at 80°C. Probe HV was set to 3.8 kV. Sample cone voltage was set to 30 V. Extractor cone was set to 5.0 V. SORI RF Burst offset frequency was 1000 Hz. Irradiation time was 1000 ms. Amplitude was 3.0 V. Hexapole absolute delay was 2500 ms. The data was analyzed by DataAnalysis Compass.

### RNA Isolation and Quantitative Reverse Transcription Polymerase Chain Reaction (qRT-PCR)

For the consistency of flavonoid content and gene expression level, all the leaves examined were cut in halves. One was for HPLC analysis, and the other was for real-time PCR analysis (**Supplementary Figure S1**). Ginkgo leaves were harvested and total RNA was extracted with a Plant RNeasy Mini Kit (Qiagen). The reverse transcript reaction was performed with ReverTra Ace qPCR RT Kit (TOYOBO) according to the manufacturer’s instructions. The transcript levels were measured by qRT-PCR using a Mx3000p QPCR System (Agilent) with iQ SYBR Green Supermix (Bio-Rad). The relative expression levels were calculated according to the 2^-ΔΔ^*^C^*_T_ method (Livak and Schmittgen, 2001; Zhang et al., 2015). Each experiment was carried out with at least three independent biological replicates. GenBank accession numbers were as follows: *GbPAL*, EU071050; *GbCHS*, AY647263; *GbF3H*, AY742228; *GbFLS*, AAS21058; *GbANS*, ACC66093; *GbMYBF2*, JQ068807; *GbGAPDH*, L26924. Primer sequences used for qRT-PCR are listed in **Supplementary Table S1**.

## Results

### The Establishment of a Culture System for Post-harvest Ginkgo Leaves

**FIGURE 1:**
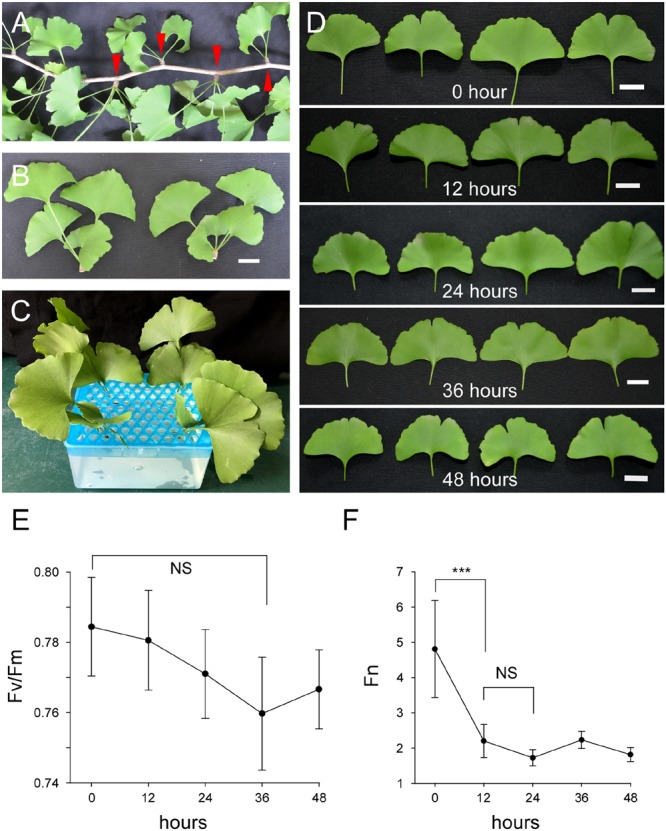
Cultured post-harvest Ginkgo leaves are able to maintain vitality for a long period of time. **(A)** A branch of Ginkgo tree with several short shoots indicated by red arrow heads. **(B)** Separated short shoots of Ginkgo trees. Bar = 2 cm. **(C)** Cultured Ginkgo leaves in MS solution. **(D)** Phenotypes of Ginkgo leaves after different periods of culture in solution. No significant changes are observed within 48 h. Bars = 2 cm. **(E)** *F*v/*F*m values of Ginkgo leaves after different periods of culture in solution. **(F)** *F*n values of Ginkgo leaves after different periods of culture in solution. Data for independent experiments are shown (mean ± SD; *n* = 10; NS, not significant *P* > 0.05; ^∗∗∗^*P* < 0.001; student’s *t*-test).

To investigate the regulation of flavonoid biosynthesis in post-harvest Ginkgo leaves, we established a culture system for these leaves. Ginkgo branches with short shoots (also known as spur shoots) were collected (**Figure 1A**). The short shoots were harvested and cultured in a growth chamber under certain conditions (**Figures 1B,C**). The leaves, which were clustered at the tip of short shoots, maintained vitality for quite a long time (**Figures 1D**–**F**). The short shoots were essential, as leaves without short shoots did not survive for such a long time in our system (**Supplementary Figure S2**). The ratio of *F*v/*F*m represents whether or not plant stress affects photosystem II. In general, the greater the plant stress, the lower the *F*v/*F*m ratio. Thus, the ratio of *F*v/*F*m is a measurement protocol that can be used for many types of plant stress (Maxwell and Johnson, 2000; Baker and Rosenqvist, 2004; Baker, 2008; Kalaji et al., 2011). We measured the *F*v/*F*m values of our cultured leaves for 48 h with an interval of 12 h. Although a slight change was observed, it was not significant (**Figure 1E**). We also measured the photosynthetic rate (Pn) of these leaves. We found that the *F*n value decreased significantly in the first 12 h, and was steady at a low value thereafter (**Figure 1F**). These results showed that post-harvest Ginkgo leaves were able to maintain vitality for a long period of time in our system, which provided us with the opportunity to carry out various treatments during this period.

### Identification of Flavonoids in Post-harvest Ginkgo Leaves

High performance liquid chromatography is widely used in both the qualitative and quantitative analysis of flavonoids (Julkunen-Tiitto et al., 2014). Here, we used HPLC to separate and identify flavonoids in post-harvest Ginkgo leaves. The HPLC fingerprint of the total extract showed several peaks, and we chose eight major peaks (indicated by 1, 2, 3, 4, 5, 6, 7, and 8) for further analysis (**Supplementary Figure S3A**). UV absorption spectrum analysis of these eight peaks showed that except for non-specific absorption near 200 nm, which was probably caused by the solvent or other impurities, all the peaks exhibited two major absorption bands in the UV region. Furthermore, six of the eight peaks (No. 1, 2, 3, 4, 5, and 6) had the first absorption band near 260 nm and the second absorption band near 350 nm, which is in accordance with the characteristic absorption spectrum of flavonoids, indicating that they were flavonoids (**Supplementary Figure S3**).

In order to confirm the presence of flavonoids, we carried out mass spectrometric analysis, and found that two peaks possibly represented rutin (quercetin 3-*O*-β-D-rutinoside) and narcissoside (isorhamnetin 3-*O*-β-D-rutinoside) (**Supplementary Figures S4**, **S5**). For a one-to-one match between the peaks and the specific types of flavonoids, we added rutin and narcissoside standards to the EGB in the HPLC experiments. As a result, we confirmed that peak No. 3 represented rutin and peak No. 5 represented narcissoside (**Supplementary Figures S6**, **S7**).

### UV-B Irradiation Induces Both the Accumulation of Flavonoids and the Expression of Corresponding Genes

**FIGURE 2:**
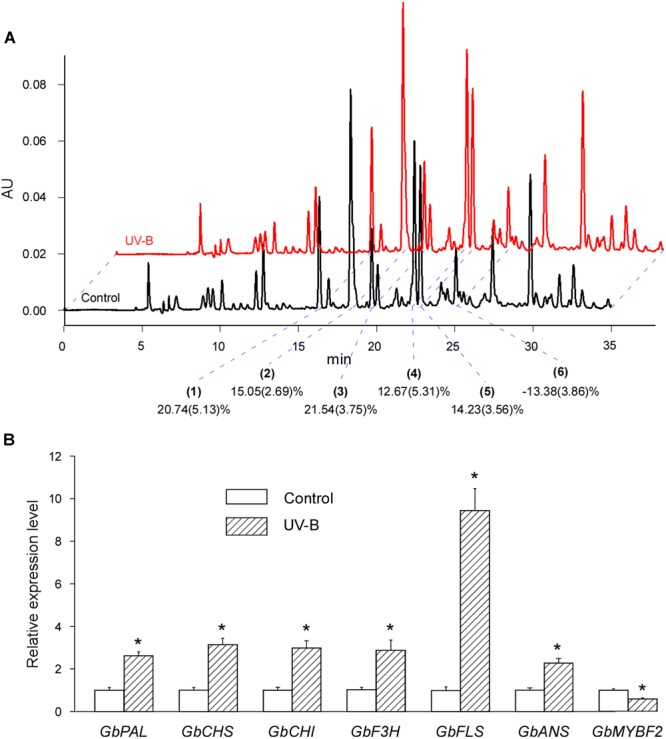
UV-B irradiation induces both the accumulation of flavonoids and the expression of corresponding genes. **(A)** Typical UV-B treated (red) and control (black) HPLC fingerprints (absorption at the wavelength of 360 nm) of the total extract of cultured post-harvest Ginkgo leaves. The peaks are justified by blue dotted lines. The main peaks (indicated by 1, 2, 3, 4, 5, and 6) are analyzed for changes after various treatments. Changes in relative flavonoid contents are marked and the SD values are in brackets. **(B)** Relative expression levels of flavonoid biosynthesis related genes induced by UV-B treatment. Asterisks indicate significant differences (*P* < 0.01; Student’s *t*-test) between UV-B treatment and control. Data for independent experiments are shown (mean ± SD; *n* = 6).

We investigated the effect of UV-B irradiation on the flavonoid contents of post-harvest Ginkgo leaves by measuring the area of the six peaks. We found that five of the six peak areas increased after UV-B irradiation (No. 1, 20.74%; No. 2, 15.05%; No. 3, 21.54%; No. 4, 12.61%; and No. 5 14.23%), while one peak area was reduced (No. 6, -13.38%). In general, UV-B irradiation induced the accumulation of flavonoids, although a minority of flavonoids had reduced contents after treatment (**Figure 2A**).

We also examined the expression of corresponding genes related to flavonoid biosynthesis. The expression of all the structural genes examined were induced by more than 2-fold, and the expression of *GbFLS* was induced by more than 10-fold. GbMYBF2 is considered a negative regulator of flavonoid biosynthesis (Xu F. et al., 2014). As expected, the expression of *GbMYBF2* decreased significantly after UV-B treatment (**Figure 2B**).

These results showed that UV-B treatment induces both the accumulation of flavonoids and the expression of corresponding structural genes. Our results also indicated that it is possible to treat post-harvest Ginkgo leaves to increase flavonoid contents.

### NaCl Treatment Induces Flavonoid Accumulation in Post-harvest Ginkgo Leaves

**FIGURE 3:**
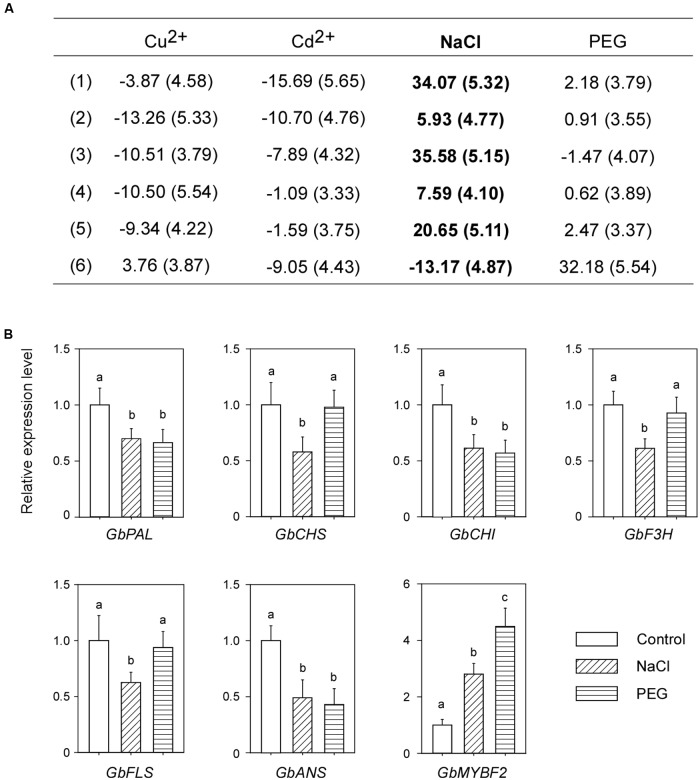
NaCl treatment induces the accumulation of flavonoids and suppresses the expression of corresponding genes in post-harvest Ginkgo leaves. **(A)** Changes in relative flavonoid contents after various treatments. Six main peaks are analyzed, the SD values are in brackets, and the changes after NaCl treatment are shown in bold. **(B)** Relative expression levels of flavonoid synthesis related genes after NaCl stress treatment. Different letters indicate significant differences (*P* < 0.01; Student’s *t*-test). Data for independent experiments are shown (mean ± SD; *n* = 6).

Using the system mentioned previously, we screened the flavonoid contents of post-harvest Ginkgo leaves under different treatments. Treatment with Cu^2+^ and Cd^2+^ did not induce the accumulation of flavonoids. Conversely, most of the flavonoids tested were reduced after these treatments. Interestingly, NaCl treatment significantly induced the accumulation of flavonoids (**Figure 3A**). To exclude the possible osmotic effect of NaCl, we also measured flavonoid contents after treatment with PEG. It was found that, compared with NaCl, PEG treatment did not change the flavonoid contents (**Figure 3A**). Our results showed that NaCl treatment induced flavonoid accumulation in post-harvest Ginkgo leaves.

### Post-harvest and Naturally Grown Ginkgo Leaves Respond Differently to NaCl Treatment

We examined the expression levels of corresponding genes related to flavonoid biosynthesis after NaCl treatment. Surprisingly, the expression of all structural genes decreased. Consistent with the decreased expression of structural genes, the expression of *GbMYBF2* increased (**Figure 3B**).

To investigate whether this unusual gene expression pattern exists in naturally grown Ginkgo trees, we treated 2-year-old Ginkgo trees with NaCl (**Supplementary Figure S8A**). The HPLC fingerprint of naturally grown Ginkgo leaves revealed that they had quite different flavonoid profiles compared with post-harvest leaves (**Supplementary Figure S8B**, compared with **Figure 2A**). Based on the time of appearance and UV absorption spectrum of the individual peaks, we determined the same six peaks that had been analyzed in the post-harvest Ginkgo leaves. NaCl treatment markedly changed the contents of flavonoids. For example, NaCl treatment induced flavonoids (corresponding to peak No. 4 and No. 6) by more than 100% (**Supplementary Figure S8C**). Importantly, the expression level of most structural genes, except *GbPAL* and *GbCHS*, increased after NaCl treatment. Interestingly, the expression of *GbMYBF2* also increased (**Supplementary Figure S8D**).

The different responses to NaCl treatment between post-harvest and naturally grown Ginkgo leaves indicated that post-harvest Ginkgo leaves may use a novel technique to accumulate flavonoids in response to NaCl treatment, which is different from naturally grown Ginkgo leaves.

### Combined UV-B and NaCl Treatment Does Not Further Increase the Flavonoid Contents in Post-harvest Ginkgo Leaves

**FIGURE 4:**
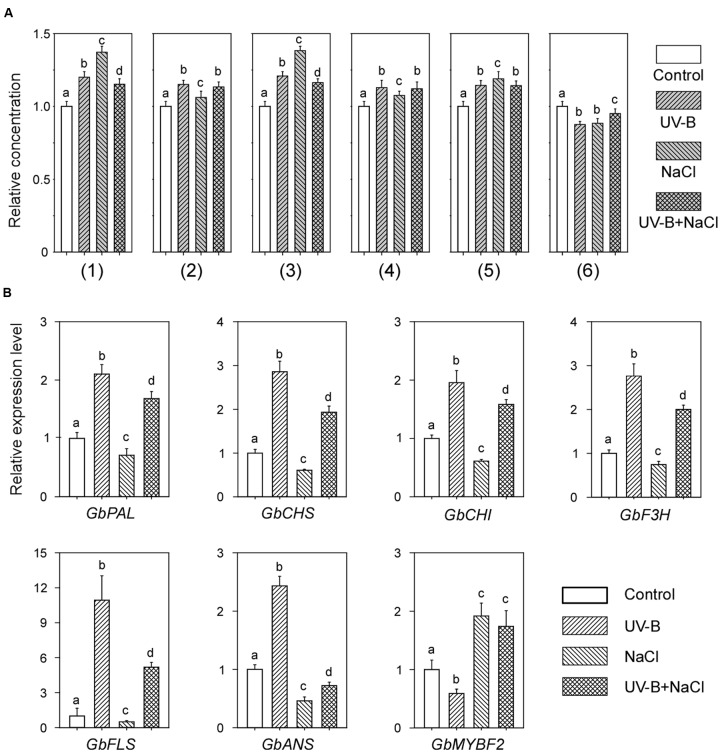
Combined UV-B and NaCl treatment have no additive effect on the flavonoid contents in post-harvest Ginkgo leaves. **(A)** Relative flavonoid contents after individual or combined treatment with UV-B and NaCl. **(B)** Relative expression levels of flavonoid biosynthesis related genes after individual or combined treatments with UV-B and NaCl. Different letters indicate significant differences (*P* < 0.01; Tukey’s test). Data for independent experiments are shown (mean ± SD; *n* = 6).

To investigate whether the combined treatment of UV-B and NaCl would further increase the flavonoid contents in post-harvest Ginkgo leaves, which is important for the pharmaceutical industry, we treated the leaves with both UV-B and NaCl. It was found that the combined treatment did not further increase flavonoid contents compared with UV-B or NaCl treatment alone. In some cases (peak No. 1 and No. 3), the combined treatment induced even fewer flavonoids than the individual treatments (**Figure 4A**).

We also examined gene expression levels in post-harvest Ginkgo leaves after combined treatment. Interestingly, following combined treatment, most of the gene expression levels were approximately the same mean values as those after UV-B and NaCl treatment alone (**Figure 4B**). These results indicated an antagonistic effect of UV-B and NaCl on the flavonoid contents in post-harvest Ginkgo leaves.

## Discussion

High performance liquid chromatography is a commonly used method for the separation of flavonoids in Ginkgo (Liu et al., 2015), and the wavelength is usually set at 360 nm to detect these flavonoids (Xu Y. et al., 2014). Considering that the raw extract may contain other different types of molecules absorbed at the examined wavelength, further confirmation was carried out by UV absorption spectrum analysis, mass spectrometric analysis, and standard confirmation. A previous report showed that different flavonoids exhibited different activities to fibril formation (Xie et al., 2014). Thus, it is more important to analyze the changes in individual flavonoids (not total flavonoids) due to different treatments. We also found many minor peaks in the HPLC fingerprints, which were probably flavonoids. However, due to the large number of natural flavonoids, which was estimated to be more than 70 (Liu et al., 2015), it is not easy to analysis the changes in these flavonoids due to different treatments. As a result, we chose six main peaks, which are probably flavonoids, for further analysis.

Flavonoids are classic UV-B-regulated compounds, and a large number of studies have shown a close relationship between UV-B irradiation and flavonoid metabolism (Zhang and Björn, 2009; Schreiner et al., 2012). In our experiment, UV-B irradiation resulted in the accumulation of flavonoids in post-harvest Ginkgo leaves. Accordingly, it also induced the expression of flavonoid structural genes and reduced the expression of a negative regulator, GbMYBF2. These results not only confirm the effectiveness of UV-B irradiation on flavonoid accumulation, but also indicate the usefulness of our research system.

Ultraviolet-B-induced flavonoids increase in post-harvest Ginkgo leaves has been investigated in other systems (Sun et al., 2010). Compared with previous research, we found that the efficiency of flavonoids increase was lower in our system. In our research, the leaves were cultured for 12 h before UV-B treatment, and the analysis was carried out immediately after UV-B irradiation. In contrast, in the system developed by Sun et al. (2010), the leaves were immediately subjected to UV-B treatment, and the analysis was carried out after an adaptation time of 24 h. This incubation before UV-B treatment may have decreased the vigor of the leaves and weakened the effectiveness of UV-B treatment, while the adaptation period after UV-B treatment may have revoked the flavonoid biosynthetic enzymes to work, as a result, the flavonoid contents were further increased. Thus, different procedures may significantly affect the results even though the treatments are the same.

We found that NaCl was an effective inducer while the others had no obvious effect on flavonoid accumulation in our experiment. To explain the ineffectiveness of heavy metal and drought treatments in the induction of flavonoid accumulation in our experiment, we propose that these stresses do induce the biosynthesis of flavonoids, but by different mechanisms. First, the responses to heavy metal and drought stresses may not be as quick as that to UV-B and NaCl. Second, considering that more than 70 types of flavonoids have been identified in Ginkgo (Liu et al., 2015), these treatments may induce other types of flavonoids instead of the “six main peaks” examined in our research. Alternatively, our results may indicate the existence of a different mechanism of stress responses specific to post-harvest Ginkgo leaves rather than naturally grown leaves.

The most unexpected result in our research was the inconsistency between the accumulation of flavonoids and the expression of corresponding structural genes in NaCl treated post-harvest Ginkgo leaves. NaCl treatment induced the accumulation of flavonoids, but reduced the expression of corresponding structural genes in post-harvest Ginkgo leaves (**Figure 3**). To explain this contradiction, we propose that Ginkgo may employ multigene families to control the flavonoid biosynthesis pathway, and the post-harvest Ginkgo leaves induced other members in the family which we did not detect in our experiment. There is growing evidence to indicate that compared with the single-copy genes in Arabidopsis, Ginkgo may employ multigene families to control each step of the flavonoid pathway, which results in a more complex network involved in flavonoid biosynthesis (Winkel-Shirley, 2001a). Southern blot analysis of *GbCHS* resulted in eight to ten bands, which indicated that a multigene family of *GbCHS* may exist in Ginkgo (Pang et al., 2005). RNA-Seq analysis of the transcriptome in Ginkgo also revealed a number of unique putative transcripts in each step of flavonoid biosynthesis, indicating the existence of multigene families in these steps (Han et al., 2015). Recently, the whole genome sequence of Ginkgo was published and a considerable expansion of genes involved in flavonoid biosynthesis was found (Guan et al., 2016). This evidence strongly supports the existence of a multigene-family-controlled flavonoid biosynthesis network in Ginkgo.

We propose that NaCl treatment of post-harvest Ginkgo leaves may cause not only salt stress, but also a special status similar to wounding. Many plants stimulate flavonoids in response to wounding caused by insect herbivory (Treutter, 2006; Anttila et al., 2010). Furthermore, the responses of Ginkgo to herbivore wounding and mechanical wounding are different. Compared with mechanical wounding, herbivore wounding resulted in a significant transmembrane potential depolarization, which was associated with increases in cytosolic calcium concentration, and H_2_O_2_ and phenolic compounds including flavonoids (Mohanta et al., 2012). In our research, the exposure of cut Ginkgo to high concentrations of NaCl disrupted the ionic equilibrium in the cells, which may trigger a response similar to that of herbivore wounding. Interestingly, it was reported that herbivore wounding of Ginkgo leaves resulted in decreased expression of *GbFLS*, although the concentration of flavonoids increased (Mohanta et al., 2012). Consistent with this response, in our experiment, NaCl treatment of post-harvest Ginkgo leaves resulted in an increased concentration of flavonoids, but decreased expression of structural genes (**Figure 3**). The results obtained following NaCl treatment of 2-year-old Ginkgo trees also showed that changes caused by NaCl in post-harvest Ginkgo leaves were not caused simply by salt stress. NaCl treatment caused increased expression levels of most structural genes in 2-year-old Ginkgo trees, while similar treatment caused decreased expression levels of structural genes in post-harvest Ginkgo leaves.

NaCl treatment of 2-year-old Ginkgo trees induced the expression of not only structural genes, but also *GbMYBF2*, which encodes a negative regulator of flavonoid biosynthesis (**Supplementary Figure S8D**). We propose that the biosynthesis of flavonoids is regulated by a complex network. The induction of both structural genes and *GbMYBF2* indicated the existence of a positive regulator in this process. MYB proteins usually form a protein complex to regulate flavonoid biosynthesis (Xu et al., 2015). It is possible that other parts of this network play a major role in the regulation of structural gene expression in NaCl treated Ginkgo trees.

Combined treatments with different factors are usually an efficient way of altering the flavonoid profile of plants (Li et al., 2012; Martínez-Lüscher et al., 2014; Neugart et al., 2014). However, in our experiment, we did not observe an additive effect on flavonoid accumulation following combined treatment with UV-B and NaCl (**Figure 4A**). We found that the induction of structural gene expression by UV-B was significantly reduced by NaCl treatment, which may decrease the portion of UV-B-induced flavonoid accumulation (**Figure 4B**). Similarly, NaCl-induced flavonoid accumulation may also be diluted by UV-B treatment. Interestingly, the antagonistic effect of UV-B and salt stresses was recently proved in poplar plants, which showed that moderate salt treatment alleviates UV-B-induced impairment in poplar plants. Similarly, the flavonoid concentration increased to the same extent in both UV-B and combined stresses in poplar plants (Ma et al., 2016). Alternatively, combined treatment with UV-B and NaCl may go beyond the physiological limit of plant cells and partially disrupt the flavonoid biosynthesis system. Exposure to mild or moderate stresses can induce active acclimation responses, while more severe conditions may cause metabolic disruptions (Jansen et al., 2008). Thus, it is important that the strength of stress treatment should be moderate in plants.

The success of producing plant flavonoids by *Escherichia coli* showed that a plant biosynthesis pathway can be established in microorganisms, which is more favorable for the pharmaceutical industry (Hwang et al., 2003). Considering the complexity of the different flavonoids in EGB, almost all institutes currently still use Ginkgo leaves for the extraction of flavonoids. Furthermore, many attempts have being made to increase the flavonoid content in Ginkgo leaves (Xu Y. et al., 2014; Wang et al., 2016). In this research, we treated post-harvest Ginkgo leaves with UV-B and NaCl. Both treatments resulted in the accumulation of flavonoids, but by different pathways. Our research indicated the distinctive status of post-harvest Ginkgo leaves, and suggested that it is necessary for us to reevaluate the effects of various stimuli in post-harvest Ginkgo leaves, not just imitate the circumstances in naturally grown plants.

## Author Contributions

JN and MX conceived and designed the experiments. JH, ZJ, XZ, LD, XY, ZS, and WX performed the experiments. JN, ZW, and MX analyzed the data. ZW contributed reagents/materials/analytical tools. JN wrote the manuscript.

## Conflict of Interest Statement

The authors declare that the research was conducted in the absence of any commercial or financial relationships that could be construed as a potential conflict of interest.

## Supplementary material

FIGURE S1 Splitting of Ginkgo leaves before analysis. **(A)** Ginkgo leaf before splitting. **(B)** The splitting and follow-up of Ginkgo leaves. Red arrow indicates tearing of the leaf.

FIGURE S2 The necessity of short shoots in the culture system. Leaves without short shoots significantly wilted 12 h after harvest. **(A)** Immediately after harvest. **(B)** 12 h after harvest. Red arrow heads indicate short shoots.

FIGURE S3 **(A)** Typical fingerprint of total extract of post-harvest Ginkgo leaves. Numbers on the peaks are candidates for UV absorption spectrum analysis. **(B)** UV absorption spectrum analysis of eight peaks selected in the fingerprint. The wavelengths of two absorption peaks are marked.

FIGURE S4 Mass spectrometric analysis of peak No. 3, which is estimated to be rutin.

FIGURE S5 Mass spectrometric analysis of peak No. 5, which is estimated to be narcissoside.

FIGURE S6 Identification of rutin in post-harvest Ginkgo leaves. **(A)** Identification of peak No. 3 (indicated by red arrow) with the rutin standard. **(B)** The ultraviolet absorption spectrum of peak No. 3. **(C)** The UV absorption spectrum of the rutin standard.

FIGURE S7 Identification of narcissoside in post-harvest Ginkgo leaves. **(A)** Identification of peak No. 5 (indicated by red arrow) with the narcissoside standard. **(B)** The UV absorption spectrum of peak No. 5. **(C)** The UV absorption spectrum of the narcissoside standard.

FIGURE S8 The responses of naturally grown Ginkgo trees to NaCl treatment. **(A)** The phenotype of Ginkgo trees used for NaCl treatment. **(B)** Typical fingerprint of the total extract of naturally grown Ginkgo leaves. Numbers on the peaks are candidates for flavonoid content analysis. **(C)** Relative area changes of the six peaks after NaCl treatment. **(D)** Relative expression levels of flavonoid synthesis related genes after NaCl treatment. Asterisks indicate significant differences (*P* < 0.01; Student’s *t*-test) between NaCl treatment and control. Data for independent experiments are shown (mean ± SD; *n* = 3).

TABLE S1 Sequences of the primers used in this research.
