# Supplementary figures and images for: NaCl Induces Flavonoid Biosynthesis through a Putative Novel Pathway in Post-harvest Ginkgo Leaves
Source: Front Plant Sci. 2017 Jun 12;8:920. doi: 10.3389/fpls.2017.00920 (PMC5466993; doi:10.3389/fpls.2017.00920)

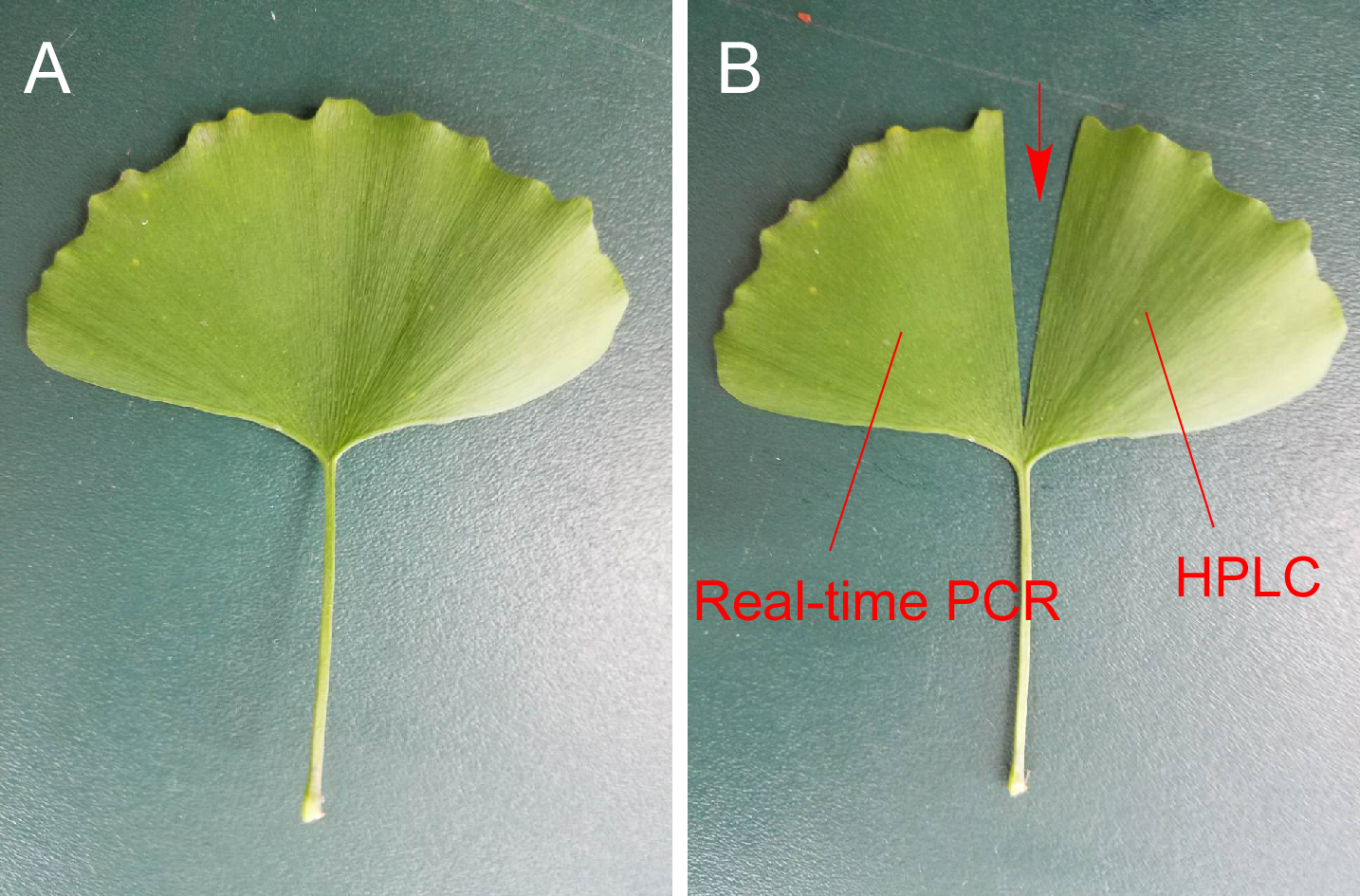

Supplement: FIGURE S1 — Splitting of Ginkgo leaves before analysis. (A) Ginkgo leaf before splitting. (B) The splitting and follow-up of Ginkgo leaves. Red arrow indicates tearing of the leaf. [file Image_1.TIF]

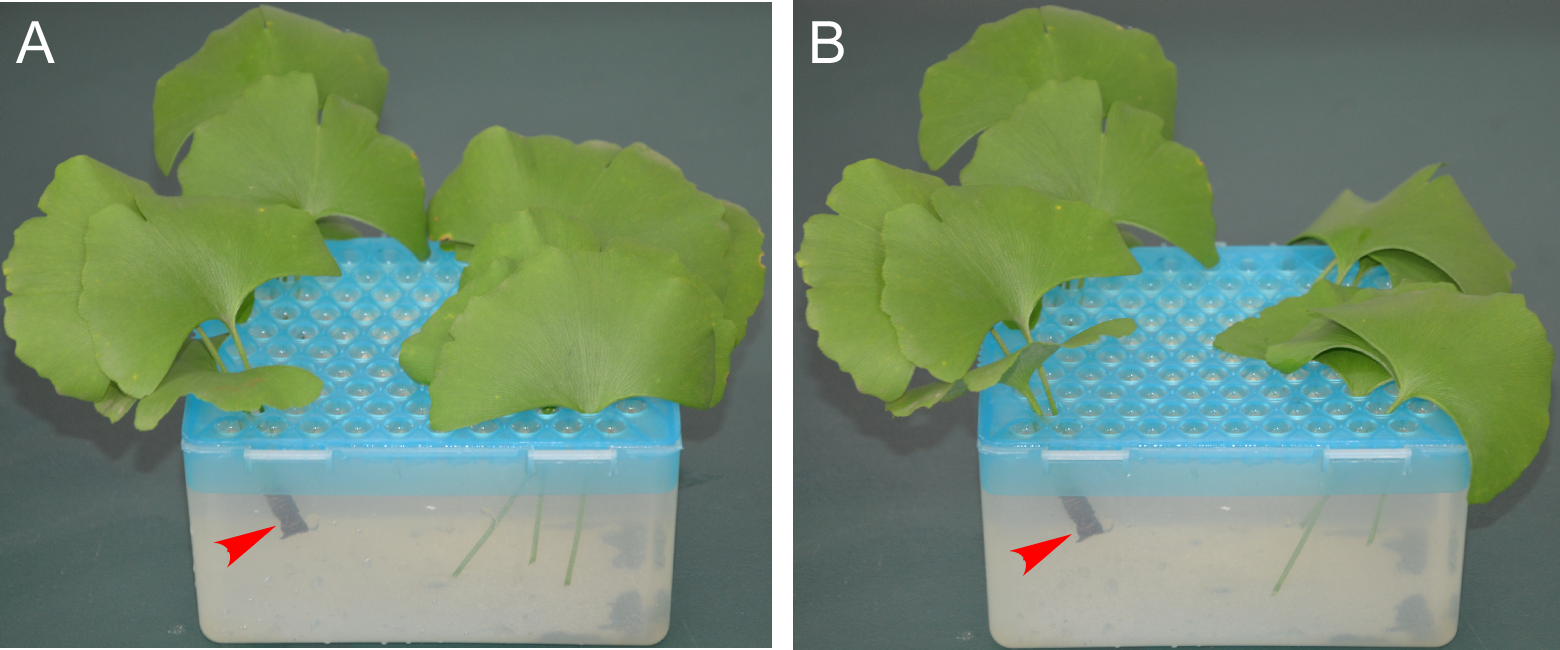

Supplement: FIGURE S2 — The necessity of short shoots in the culture system. Leaves without short shoots significantly wilted 12 h after harvest. (A) Immediately after harvest. (B) 12 h after harvest. Red arrow heads indicate short shoots. [file Image_2.TIF]

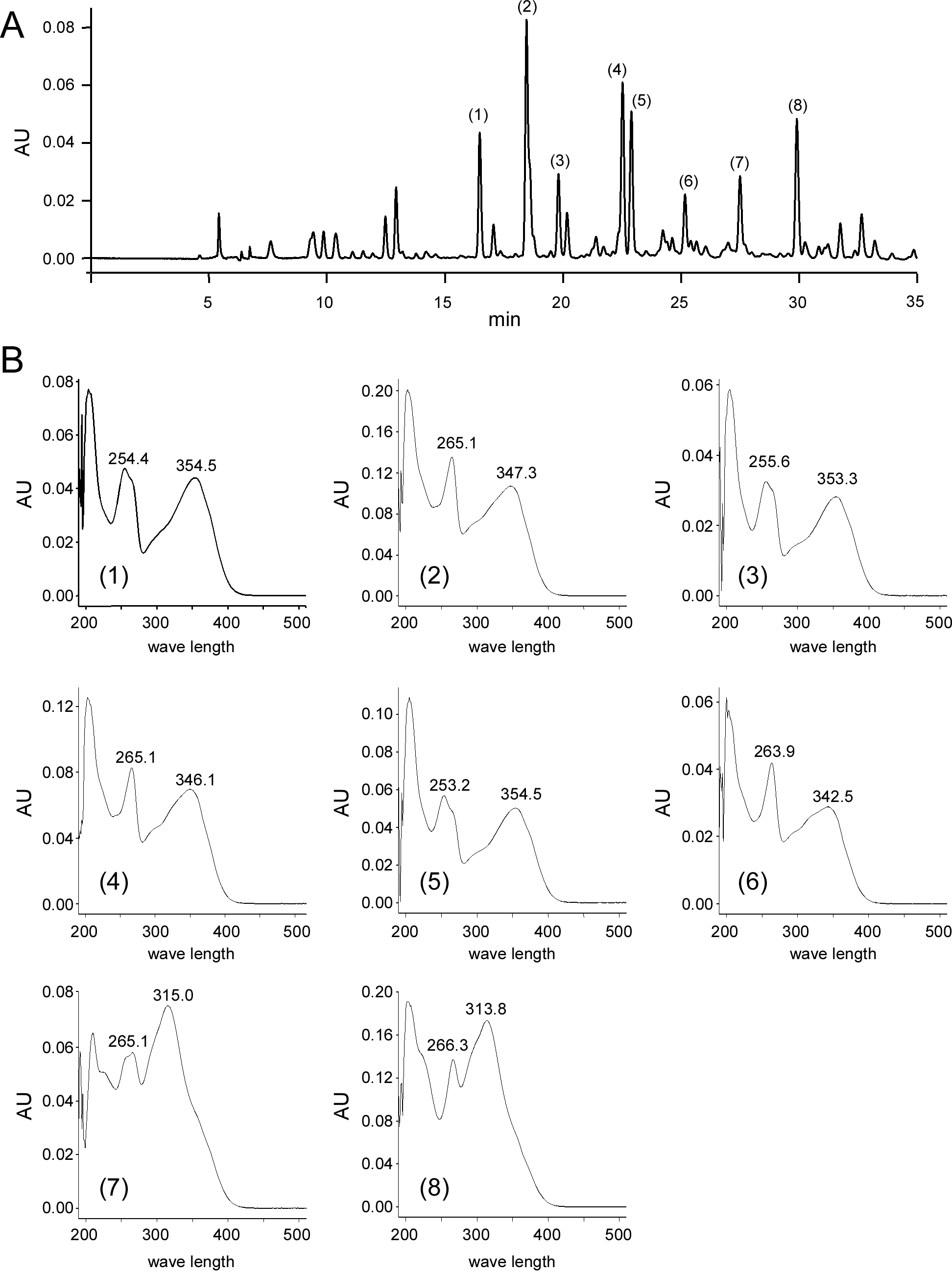

Supplement: FIGURE S3 — (A) Typical fingerprint of total extract of post-harvest Ginkgo leaves. Numbers on the peaks are candidates for UV absorption spectrum analysis. (B) UV absorption spectrum analysis of eight peaks selected in the fingerprint. The wavelengths of two absorption peaks are marked. [file Image_3.TIF]

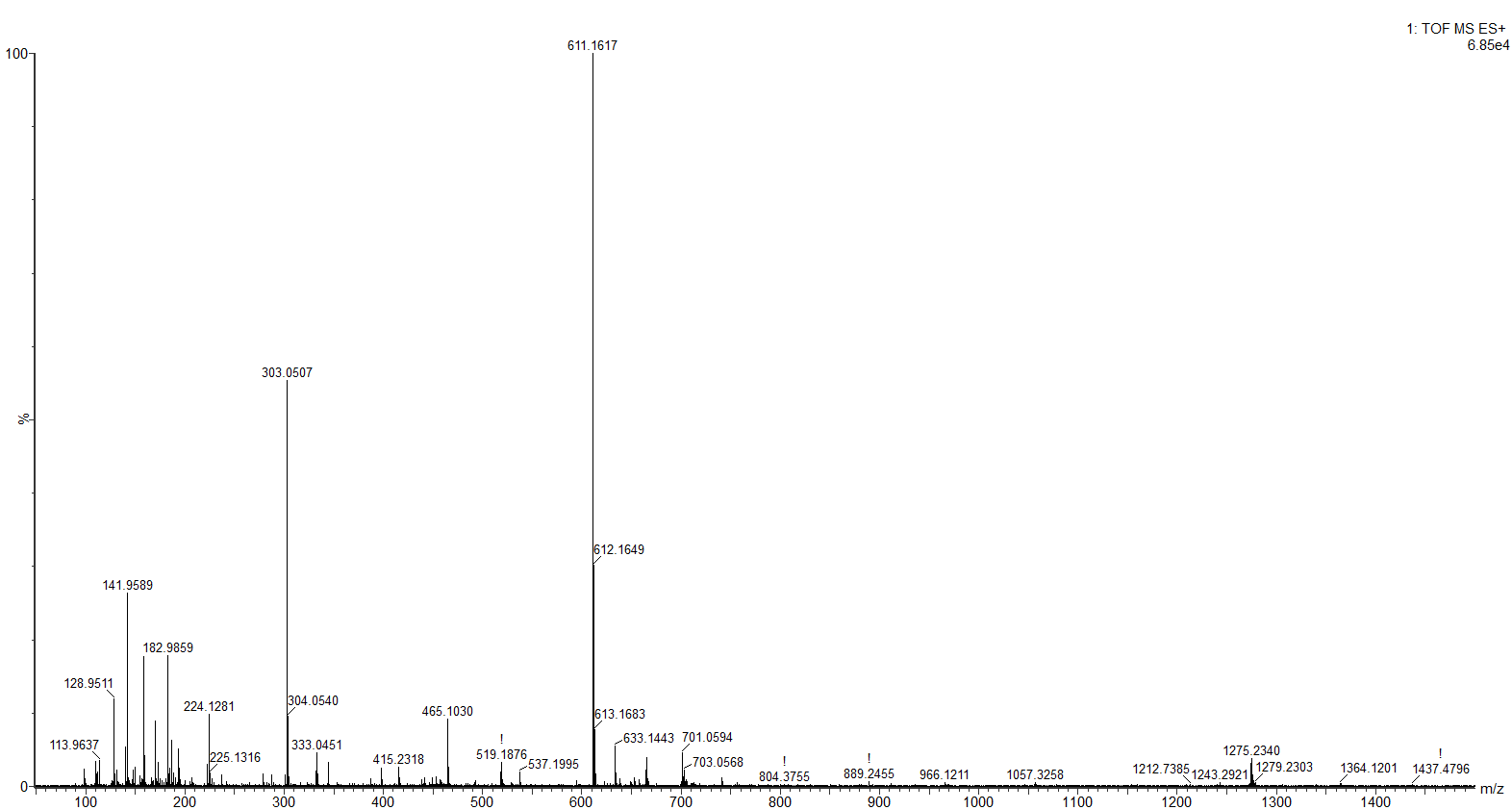

Supplement: FIGURE S4 — Mass spectrometric analysis of peak No. 3, which is estimated to be rutin. [file Image_4.TIF]

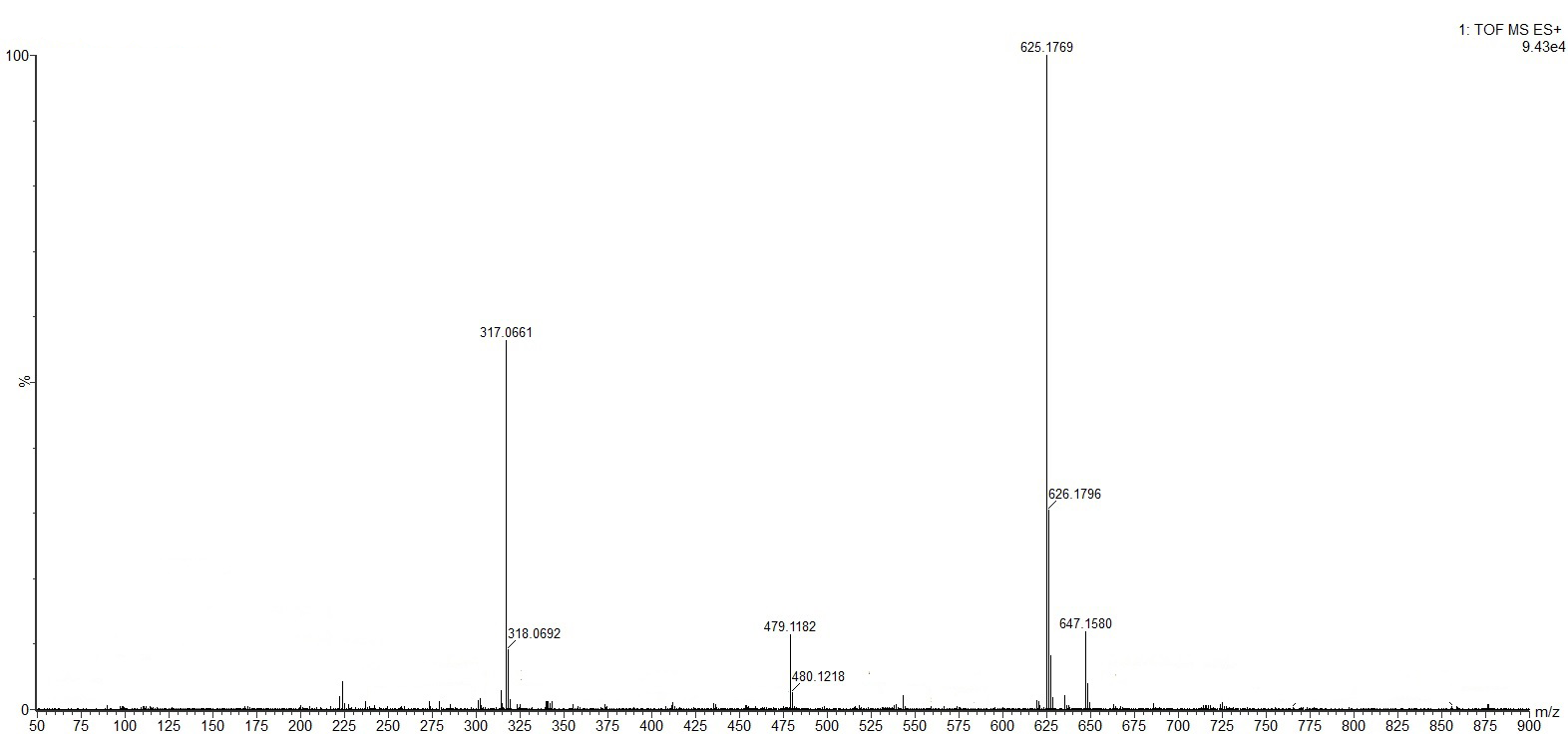

Supplement: FIGURE S5 — Mass spectrometric analysis of peak No. 5, which is estimated to be narcissoside. [file Image_5.TIF]

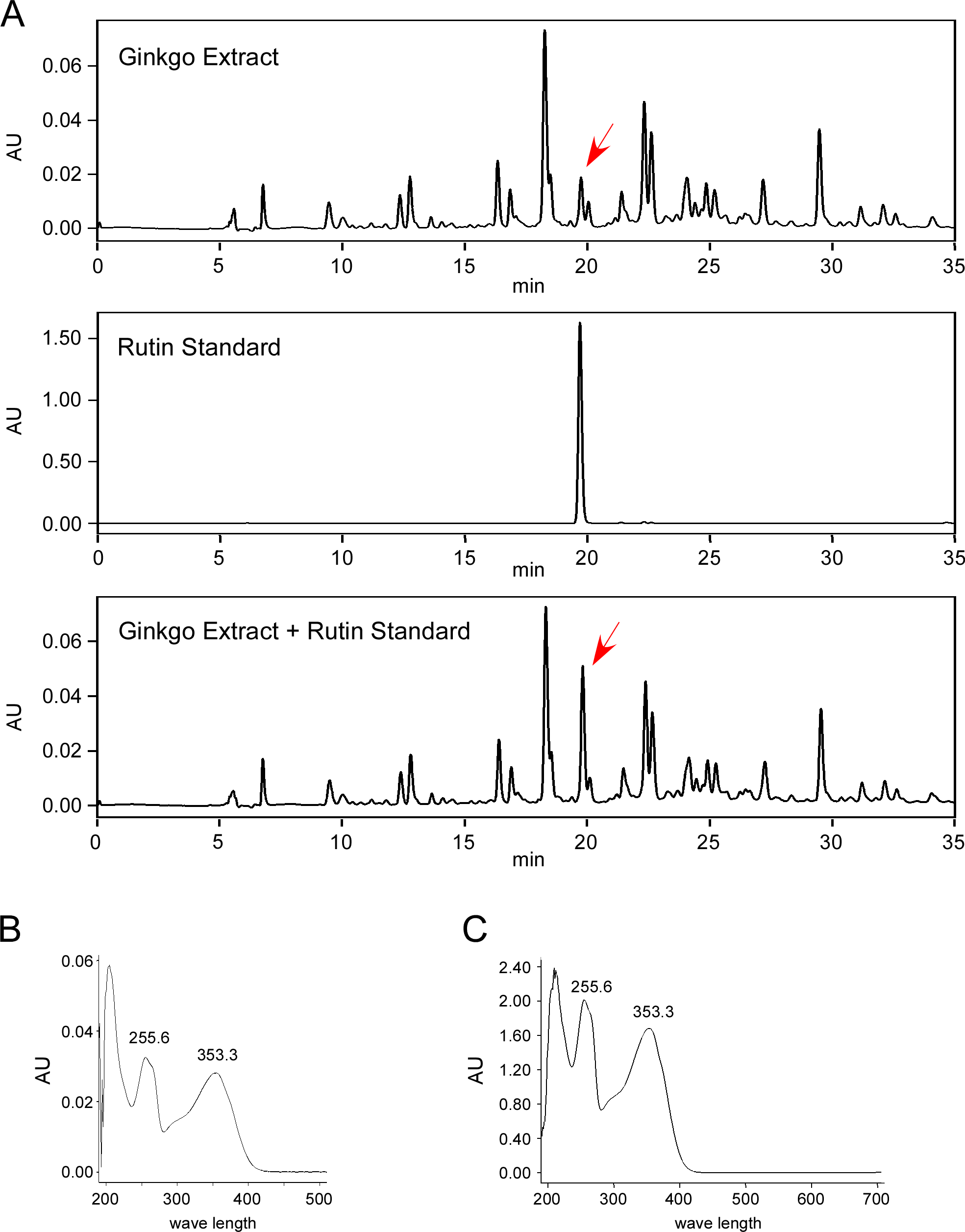

Supplement: FIGURE S6 — Identification of rutin in post-harvest Ginkgo leaves. (A) Identification of peak No. 3 (indicated by red arrow) with the rutin standard. (B) The ultraviolet absorption spectrum of peak No. 3. (C) The UV absorption spectrum of the rutin standard. [file Image_6.TIF]

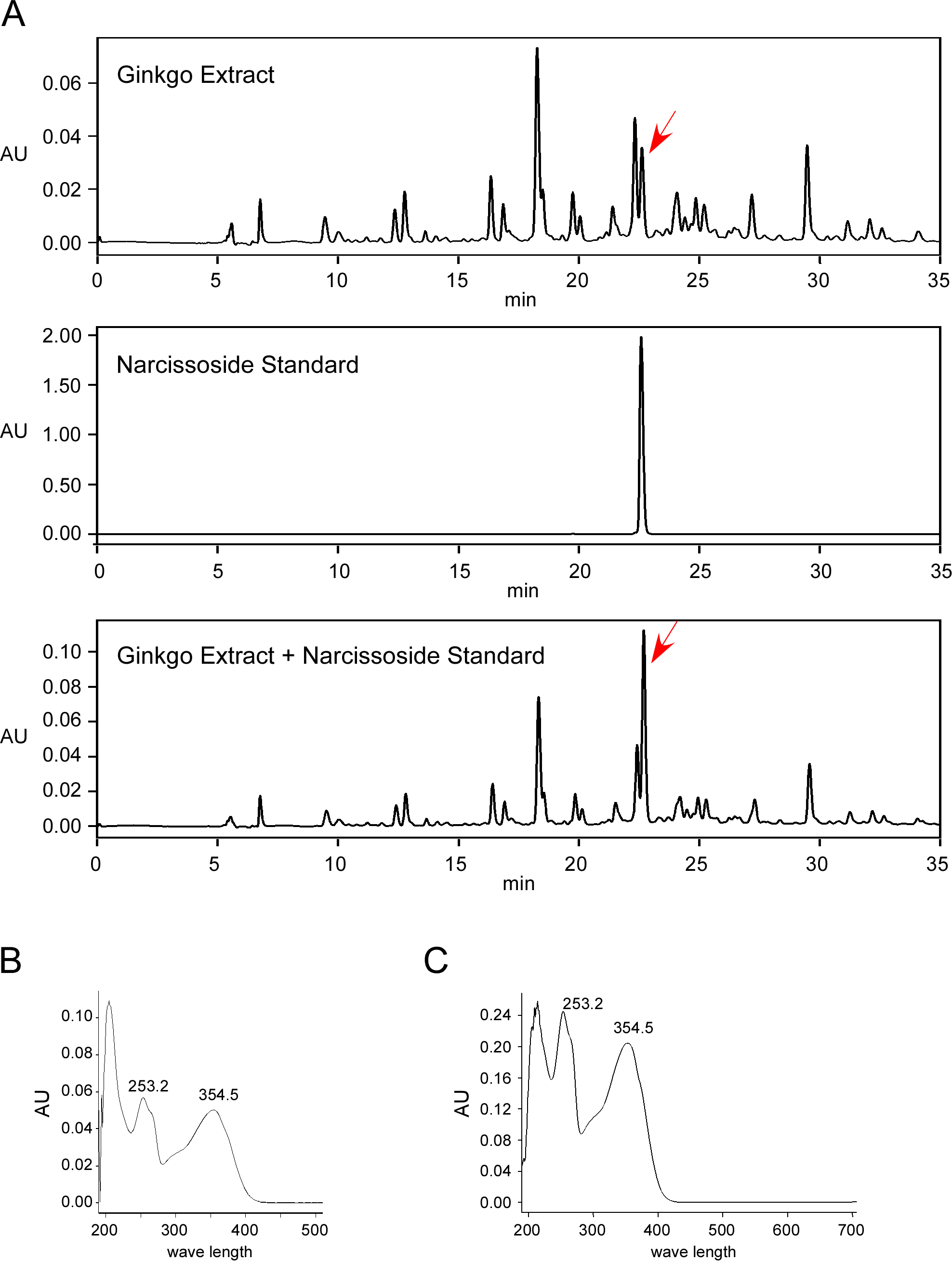

Supplement: FIGURE S7 — Identification of narcissoside in post-harvest Ginkgo leaves. (A) Identification of peak No. 5 (indicated by red arrow) with the narcissoside standard. (B) The UV absorption spectrum of peak No. 5. (C) The UV absorption spectrum of the narcissoside standard. [file Image_7.TIF]

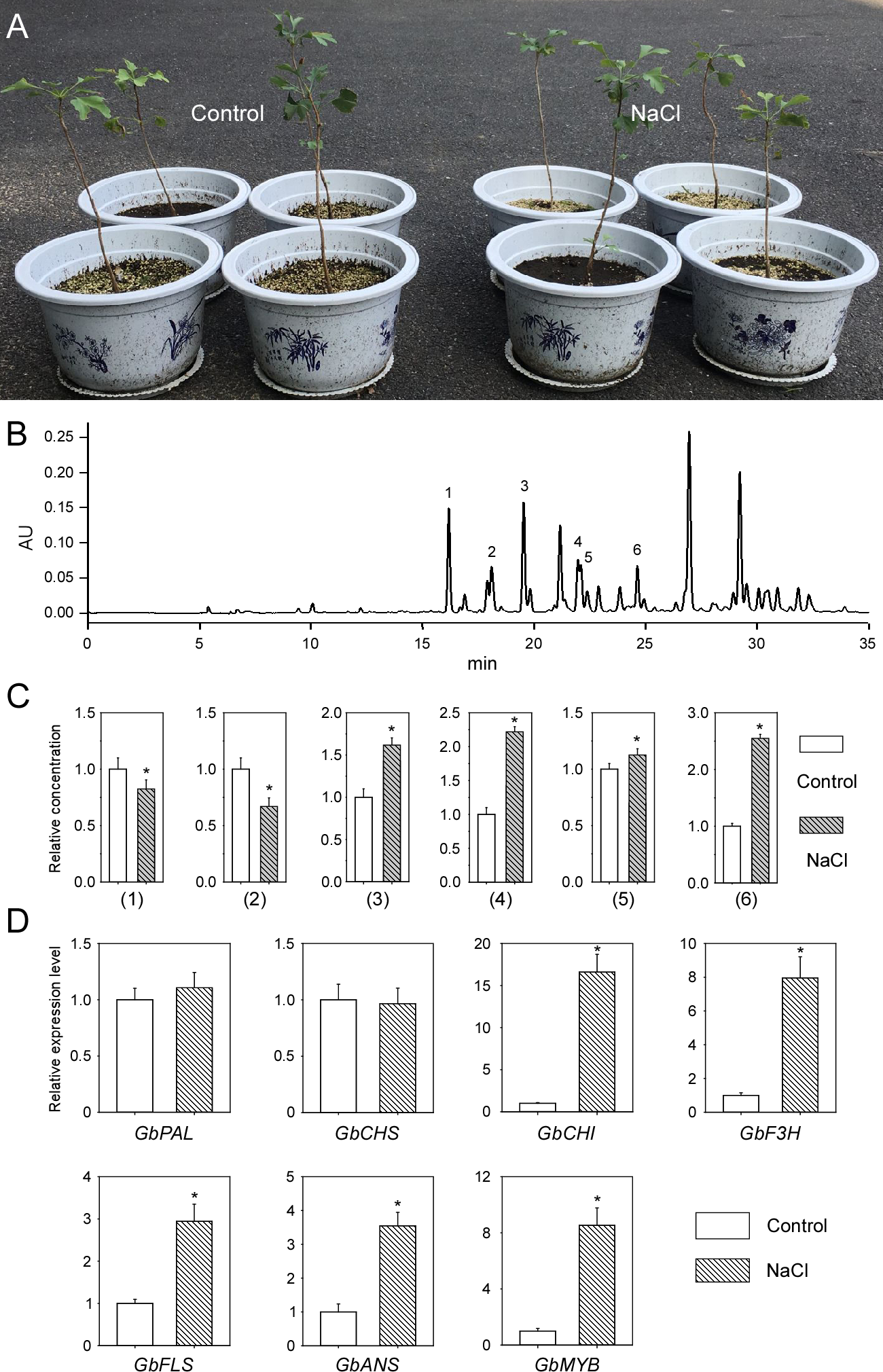

Supplement: FIGURE S8 — The responses of naturally grown Ginkgo trees to NaCl treatment. (A) The phenotype of Ginkgo trees used for NaCl treatment. (B) Typical fingerprint of the total extract of naturally grown Ginkgo leaves. Numbers on the peaks are candidates for flavonoid content analysis. (C) Relative area changes of the six peaks after NaCl treatment. (D) Relative expression levels of flavonoid synthesis related genes after NaCl treatment. Asterisks indicate significant differences (P < 0.01; Student’s t-test) between NaCl treatment and control. Data for independent experiments are shown (mean ± SD; n = 3). [file Image_8.TIF]
